# Dengue virus and the host innate immune response

**DOI:** 10.1038/s41426-018-0168-0

**Published:** 2018-10-10

**Authors:** Naoko Uno, Ted M. Ross

**Affiliations:** 1 Center for Vaccines and Immunology, University of Georgia, Athens, GA USA; 2 Department of Infectious Diseases, University of Georgia, Athens, GA USA

## Abstract

Dengue virus (DENV) is a mosquito-borne *Flavivirus* that is endemic in many tropical and sub-tropical countries where the transmission vectors *Aedes* spp. mosquitoes resides. There are four serotypes of the virus. Each serotype is antigenically different, meaning they elicit heterologous antibodies. Infection with one serotype will create neutralizing antibodies to the serotype. Cross-protection from other serotypes is not long term, instead heterotypic infection can cause severe disease. This review will focus on the innate immune response to DENV infection and the virus evasion of the innate immune system by escaping recognition or inhibiting the production of an antiviral state. Activated innate immune pathways includes type I interferon, complement, apoptosis, and autophagy, which the virus can evade or exploit to exacerbate disease. It is important to understand out how the immune system reacts to infection and how the virus evades immune response in order to develop effective antivirals and vaccines.

## Dengue virus

### Epidemiology

Dengue virus (DENV) is the most prevalent arbovirus worldwide, found in over 100 tropical and sub-tropical countries^1^. It is transmitted mainly by the *Aedes aegypti and Aedes albopictus* mosquitoes. Over half of the global population is at risk for dengue infection, with 100 million symptomatic cases being reported every year^2^. There are four genetically distinct serotypes of the virus, DENV1–4^3^. Due to the antigenic differences between the serotypes, infection with one serotype will confer long-lasting immune protection against that serotype only, while cross-protection against other serotypes are short term. In endemic countries, more than one serotype of DENV circulates^2^.

Primary infections may cause a rash and fever, but many infections are asymptomatic. Secondary infections, however, are known to cause severe disease, specifically after a heterotypic infection^4^. The exact cause of this is unknown, but the phenomenon of antibody-dependent enhancement (ADE) may cause increased pathogenicity and virulence^5^. ADE occurs when antibodies from a previous heterotypic infection do not neutralize a secondary infection with a different subtype but still bind to viral proteins. This creates a virus–antibody complex phagocytosed by cells that are not usually infected via Fcγ receptors, specifically monocytes via FcγIIa receptor^6^. This results in increased viremia and pathology. Severe disease is seen in only 1% of DENV cases; however, mortality in severe cases can have a rate of up to 20%^4^

### The virion

DENV is part of the *Flaviviridae* family, which also includes Zika, Yellow Fever, Japanese Encephalitis, and West Nile viruses. They are enveloped and spherical, with a positive-sensed and single-stranded RNA (ssRNA) genome that encodes one open reading frame with three structural (capsid, precursor membrane (prM), and envelope) and seven nonstructural (NS) proteins (NS1, NS2a, NS2b, NS3, NS4a, NS4b, and NS5). The genome is approximately 11,000 kb in length, containing a type I cap at the 5′ end and lacking a 3′ poly(A) tail^7^. The virus structure consists of a well-organized outer shell with an icosahedral symmetry, a lipid bilayer, and a poorly ordered nucleocapsid core that encapsulates the RNA genome^8^.

Out of the three structural proteins, the envelope glycoprotein (E) is the main target for neutralizing antibodies and is responsible for receptor binding and fusion^7^. It is a class II fusion protein, with 90 E dimers lying flat on the surface of the virion^8^. The membrane protein sits below the E protein on the surface of the mature virion. Immature virus particles, on the other hand, has a prM protein that forms protruding trimers with E, which creates a “spiky” appearance instead of the smooth, icosahedral structure of the mature form^9^. The capsid protein is found below the outer protein shell and the lipid bilayer. It is not as well ordered as the other structural proteins, and it is difficult to discern the viral RNA from the capsid during cryo-electron microscopy imaging^10^.

**Fig. 1:**
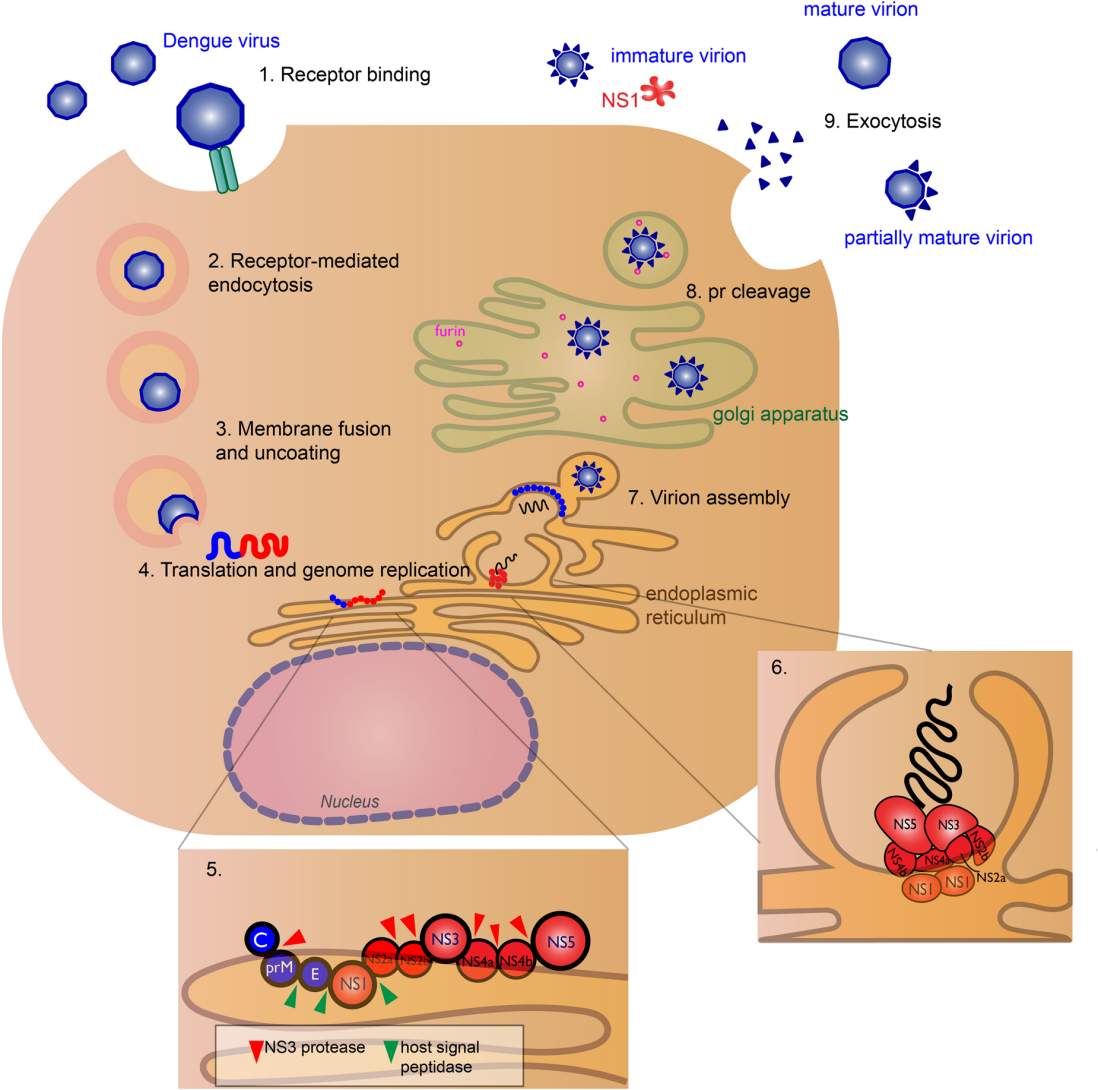
The viral life cycle of dengue virus (DENV). The virus binds to host cell receptors (exact receptors are unknown) (1) and enters the host cell (DENV permissive cells include keratinocytes, dendritic cells, endothelial cells, fibroblasts, macrophage, and mast cells), via receptor-mediated endocytosis (2). Acidification of the endosome induces conformational change of the E glycoprotein causing the virus to fuse with the endosomal membrane and release its genomic RNA material into the cytoplasm (3). DENV RNA translation and replication occur at the endoplasmic reticulum (ER) (4). The host ribosome directly translates the genomic RNA into a polyprotein, where host and viral proteases cleave the nascent protein into structural (blue) and nonstructural (red) proteins (5). RNA replication occurs in virus-induced membrane vesicles by the viral replication complex, with the transmembrane NS2a, NS2b, NS4a, and NS4b proteins acting as the scaffold (6). The viral genome is packaged into the immature virus particles during assembly (7). These particles are transported through the Golgi apparatus, where host furin-like proteases cleave the prM peptide (8), and the nascent viral particles exit the cell via exocytosis as fully mature virions (9). Some pr peptides are not cleaved resulting in immature, non-infectious virions or partially mature virions. The soluble NS1 hexamer is also secreted^12^

The NS proteins are responsible for viral replication and host immune evasion. The exact roles of NS1 and the transmembrane proteins NS2a, NS2b, NS4a, and NS4b are not well characterized. NS1 is dimeric in early stages of infection and secreted in hexameric form in later stages^11,12^. The NS1 dimer is located on the lumen side of the ER, yet it plays an essential role in viral RNA replication, since deletion of NS1 from the viral genome inhibits replication^13^. Through transmembrane interaction with NS4a and NS4b, NS1 will help form vesicles for virus replication, called the viral replication complex (RC), and colocalize with double-stranded RNA (dsRNA)^13,14^ (Fig. 1). It also modulates infectious virus particle production by interacting with structural proteins prM/E^15^. NS4a plays a role in membrane alteration, in order to form the RC^16^. NS2a is crucial for viral RNA synthesis and virion assembly^17^. NS2b binds to NS3 and forms the functional NS3 protease^18^. NS4b interacts with the NS3 helicase domain^19^.

NS3 and NS5 are the best characterized out of the NS proteins, both having enzymatic activity essential for viral replication. NS3 functions as the viral protease and helicase. The N-terminal domain of the NS3 is essential for protease activity^18^, cleaving the viral polypeptide into structural and nonstructural proteins. The C-terminal end contains the helicase domain^19^, necessary for unwinding the RNA duplex during replication. Cofactor NS2b is necessary for the protease to be functional. NS5 is the largest and most conserved of the *Flavivirus* proteins^7^. It functions as the viral RNA-dependent RNA polymerase (RdRp), and the N-terminal domain contains the methyltransferase, which is responsible for 5′-RNA capping of the new viral genomes^20^.

### Viral life cycle

DENV is spread to humans from an infected mosquito. The exact cell types and binding receptors on human cells are also unknown. Many putative receptors have been proposed as candidates, including receptors such as heparan sulfate, glycosphingolipid nLc_4_Cer, DC-SIGN, mannose, CD14, and HSP70/90^21^. Cells that are permissive for DENV infection are dendritic cells (DCs), endothelial cells, fibroblasts, keratinocytes, macrophage, mast cells, and monocytes^22^.

After receptor binding to the DENV E glycoprotein, the virus will enter the cell via clathrin-mediated endocytosis and a drop in endosomal pH triggers conformational change of the virion that leads to membrane fusion and release of viral genome into the cytoplasm^23^. Replication occurs in association with virus-induced cellular membrane structures that form the RC^24^. The positive-sensed RNA genome can be immediately translated to a polyprotein by the host ribosome^25^. The polyprotein is cleaved by host and viral proteases, with structural prM and E proteins assembling in the ER lumen and the NS proteins localizing in virus-induced membrane vesicles for RNA synthesis^26^.

After protein translation and genome replication, the virus is assembled and transported to the golgi apparatus, where the prM is cleaved by host furin protease to form the mature virion^26^. The mature, infectious virion is released by exocytosis^27^. However, prM cleavage is not efficient and immature and partially mature virions can be secreted^28^. Hexameric form of NS1 is also secreted^12^ (Fig. 1). Immature particles are non-infectious by themselves, though non-neutralizing antibodies to prM can aid in virus uptake in Fc-receptor-bearing cells^29^.

## Innate immune responses

### Viral sensing

DENV is transmitted to people via a mosquito bite. Following infection, the virus initially replicates in skin cells, such as keratinocytes and Langerhans cells^22^. This will trigger a variety of host innate immune responses. Innate immune cells are the first to respond to infection by using pattern recognition receptor (PRR) recognizing pathogen-associated molecular patterns^30,31^. These immune cells include DCs, macrophages, and monocytes. PRR recognition will trigger production of cytokines and chemokines, which induce an antiviral state. The PRRs that are associated with DENV recognition are cytoplasmic retinoic acid-inducible gene I (RIG-I) and melanoma differentiation-associated protein 5 (MDA5), along with endosomal Toll-like receptor 3 (TLR3) and TLR7^32,33^. Activation of these receptors by DENV recognition induces type 1 interferon (IFN) responses.

**Fig. 2:**
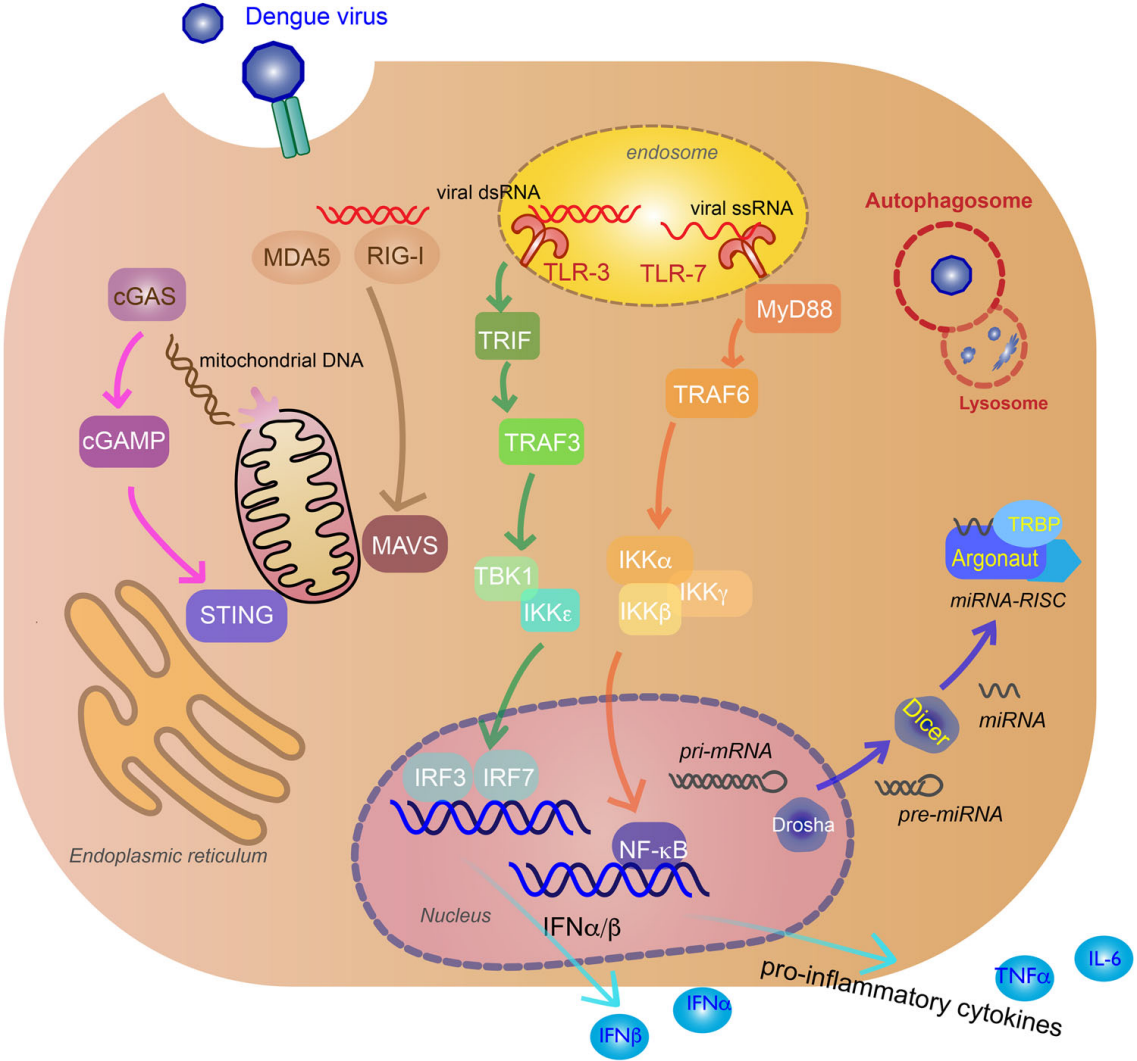
Innate immune response to DENV infection. Recognition of viral genomes by cytoplasmic retinoic acid-inducible gene I (RIG-I) and melanoma differentiation-associated protein 5 (MDA5) trigger mitochondrial antiviral signaling (MAVS) activation that lead to TANK-binding kinase 1 (TBK1), IκB kinase-ε (IKKε) induction of interferon regulatory factor 3 (IRF3), and IRF7 (tan arrows). Viral genome recognition by endosomal toll-like receptor 3 (TLR3) (green arrows) and TLR7 (orange arrows) will activate TIR-domain-containing adapter-inducing IFNβ (TRIF) and myeloid differentiation primary response gene 88 (MyD88) signaling pathways, inducing IRF3/IRF7 and inhibitor of nuclear factor-κB kinase (IKK)α/IKKβ/IKKγ, and activate nuclear factor-κB (NF-κB) to produce IFNα/β and pro-inflammatory cytokines. Virus-induced mitochondria damage activates the cyclic GMP-AMP synthase (cGAS) (pink arrows) and stimulator of interferon gene (STING) pathway to induce IFNα/β production via IRF3 and IRF7. MicroRNAs (miRNAs) associate with RNA-induced silencing complex (RISC) in the cytoplasm to target viral RNA for inhibition or degradation. miRNA biogenesis starts in the nucleus as pri-miRNA and processed by Drosha into pre-miRNA. Pre-miRNAs are transported to the cytoplasm and cleaved by Dicer to produce mature miRNAs^100^. Argonaute (Argo) and TAR RNA-binding protein (TRBP) are proteins essential to the formation of RISC (blue arrows). Double membrane vacuoles, called autophagosomes, will engulf foreign cytoplasmic material and fuse with the lysosome for degradation, inhibiting virus replication (red)

RIG-I and MDA5 are RIG-I-like receptors (RLRs), located in the cytoplasm of a variety of cells, including myeloid, epithelial, and central nervous system. They sense phosphate-containing RNA in the cytoplasm and long dsRNA^30^. Thus, they are an essential part of the innate immune response against virus, sensing viral replication in the cytoplasm. The exact RNA ligands on DENVs that these receptors recognize are unknown. Following virus recognition, the RLRs translocate to the mitochondrial membrane and activate mitochondrial antiviral signaling (MAVS) protein found on the surface of the mitochondria via the caspase activation recruitment domains (CARD) of the RLRs and MAVS. This leads to activation of TANK-binding kinase 1 (TBK1), IκB kinase-ε (IKKε), phosphorylating IFN regulatory factors (IRF3), and IRF7, which enter the nucleus to induce production of type I IFNs such as IFN-β^34^ (Fig. 2).

Another type of PRR that recognizes viruses are the toll-like receptors (TLRs). Two TLRs critical in the innate response to DENV infection are TLR3 and TLR7. TLR3, which is the primary TLR for DENV, recognizes dsRNA in endosomal compartments and TLR7 recognizes ssRNA in DC endosomal compartments^35^. TLR3 activation causes phosphorylation of TIR-domain-containing adapter-inducing IFNβ, interacting with TNF-receptor-associated factor 3 (TRAF3) and TBK1/IKKε to induce IFNα/β-stimulating genes (ISGs) and chemokines^31^. TLR3 acts synergistically with RIG-I and MDA5 in producing an antiviral state against DENV infection^32^. TLR7 recognition of ssRNA, including DENV genomic fragments, uses the myeloid differentiation primary response gene 88-dependent signal pathway to induce pro-inflammatory cytokines by recruiting TRAF6 to activate inhibitor of nuclear factor-κB kinase (IKK)α/IKKβ/ IKKγ, and activate nuclear factor-κB (NF-κB)^33^ (Fig. 2).

The cyclic GMP-AMP synthase (cGAS) and stimulator of IFN gene (STING) pathway is also activated during DENV infection by the cGAS PRR, despite the fact that this pathway recognizes cytoplasmic DNA^36^. DENV damages the mitochondria by inducing swelling and other morphological changes^37^; thus, the cGAS-STING is activated by mitochondrial DNA (mtDNA) released in the cytosol^38^. After mtDNA sensing, the cGAS nucleotidyl transferase produces second-messenger cyclic GMP-AMP (cGAMP) that binds to STING, leading to activation of TBK1, phosphorylation of IRF3, and production of type I IFNs^39^ (Fig. 2). This release of mtDNA has been shown to activate TLR9, an endosomal PRR that recognizes DNA containing nonmethylated CpG motifs, in human DCs in vitro^40^.

### Type I IFN response

**Fig. 3:**
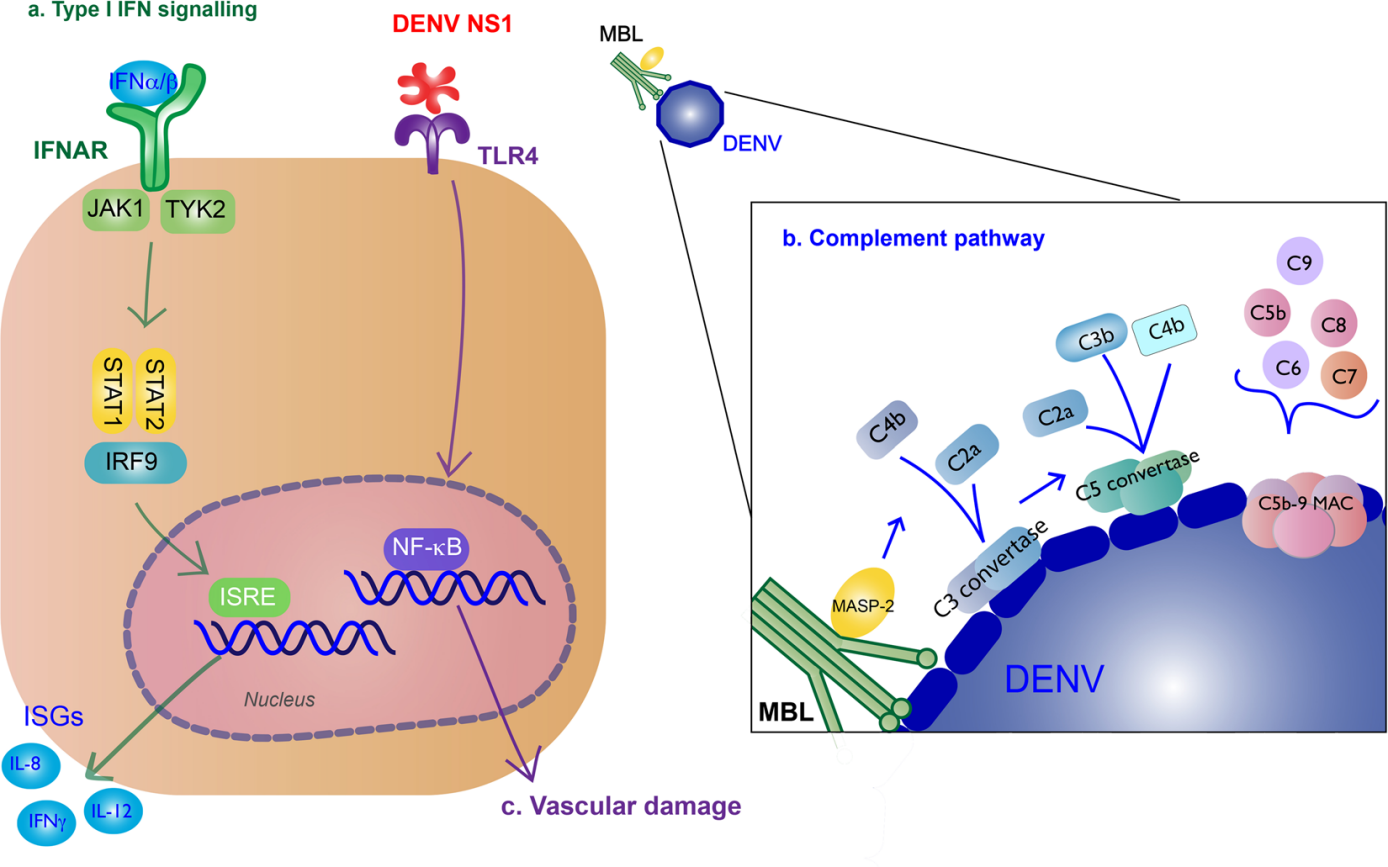
Type I IFN response and complement activation from DENV infection. **a** Type I IFN signaling (green): Binding of type I IFN to IFNα/β receptors (IFNARs) stimulates IFN-stimulated gene (ISG) expression that results in antiviral activity. These cytokines bind to on the surface of nearby or infected cells, activating the Janus kinase (JAK)/signal transducer and activator of transcription (STAT) pathway. JAK1 and tyrosine kinase 2 (TYK2) lead to phosphorylation and dimerization of STAT1 and STAT2, which forms a complex with interferon regulatory factor 9 (IRF9). The complex will translocate to the nucleus where they induce transcription of ISGs by the IFN-stimulated response element (ISRE). **b** Complement pathway (blue): Recognition of DENV by the mannose-binding lectin (MBL) complex will induce complement activation. Cleavage of C4 and C2 by MBL-associated serine protease-2 (MASP-2) make the C3 convertase and initiates the classical complement cascade, including the formation of C5 convertase and the C5b-9 membrane attack complex (MAC) to induce lysis, recruitment of phagocytes, and inflammation. **c** NS1 binding to TLR4 will induce vascular damage (purple)

Production of type I IFNs inhibits DENV infection of other monocytes^41^. These cytokines bind to IFNα/β receptors (IFNARs) on the surface of nearby or infected cells, activating the Janus kinase (JAK)/signal transducer and activator of transcription (STAT) pathway, and producing ISGs to further promote antiviral activity^42^. IFNα/β cytokines bind to IFNAR, activating JAK1 and tyrosine kinase 2, leading to the phosphorylation and dimerization of STAT1 and STAT2, which forms a complex with IRF9. The complex will translocate to the nucleus where they induce transcription of ISGs by the IFN-stimulated response element (Fig. 3).

### Complement system response

The complement system is also an important part of innate immune response to the virus. The mannose-binding lectin (MBL) pathway induces neutralizing protection against DENV. MBL binds to surfaces containing mannose glycans, and this protein will recognize the surface of DENV^43^. Recognition of DENV by the MBL complex will induce cleavage of C4 and C2 by MBL-associated serine protease-2 and deposit C4b and C2a on the surface of the virion, making the C3 convertase^44^. The following classical complement cascade includes the formation of C5 convertase and the C5b-9 membrane attack complex (MAC) to induce lysis, recruitment of phagocytes, and inflammation^45^ (Fig. 3b).

### Other innate immune responses

RNA interference (RNAi) for antiviral defense in plants and invertebrates have been well characterized, including the DENV vector *Aedes aegypti*^46^. There is also evidence of vertebrate systems using the RNAi to inhibit virus infection, though it is not as well understood. RNAi is a sequence-specific process to regulate gene expression mediated by small interfering RNA (siRNA) or microRNA (miRNA). Dicer will create siRNA from long dsRNA, while both Drosha and Dicer are needed to create miRNA. These small non-coding RNAs will associate with RNA-induced silencing complex (RISC) in the cytoplasm to target mRNA for inhibition or degradation by Argonaute (Argo) proteins. During viral infection of mammalian hosts, viral RNA is recognized by the miRNA-RISC assembly and targeted for silencing or degradation^47^ (Fig. 2). Knockdown of essential components of RNAi (Dicer, Drosha, Argo1, Argo2) resulted increased DENV viral titer in human cell line Huh7, implicating RNAi as a regulator of DENV replication^48^. There have been a number of cellular miRNA that are modulated during DENV infection, such at let-c, miRNA-30e*, and miRNA-126-5p^49^.

Autophagy is a natural cellular process to maintain homeostasis, regulating cell degradation usually in response to starvation^50^ and disease^51^. Double membrane vacuoles, called autophagosomes, will engulf cytoplasmic material and fuse with the lysosome for degradation. Thus, autophagy is an important system to clearing the host of foreign pathogens such as viruses. Autophagy has been shown to be activated in DENV infections and has antiviral or pro-viral activity depending on cell type. Autophagy inhibits replication in monocytes, specifically under ADE conditions, which make these cells highly susceptible to infection^52^. Reticulophagy, a selective autophagy process for ER homeostasis, reduces DENV via its FAM134B receptor in endothelial cells^53^. In liver cells, autophagy has a pro-viral effect. DENV blocks the autophagosome from fusing with the lysosome, and instead uses the vacuoles for replication^54^, assembly and maturation^55^, and evading neutralizing antibodies during transmission^56^.

Apoptosis is a highly regulated process of self-destruction that cells undergo in response to stimuli such as redundant or dangerous cells like tumors or pathogen-infected cells. There are two main apoptotic pathways, the intrinsic (or mitochondrial) and extrinsic, though the two are linked and converge at the execution phase, where the cell undergoes DNA fragmentation, degradation of the cytoskeleton, and formation of apoptotic bodies that are ultimately engulfed by surrounding phaocytes^57^. DENV proteins have been shown to activate apoptosis inside infected cells. The capsid protein nuclear localization interacts with death-associated protein 6 and triggers Fas-mediated apoptosis in liver cells^58^. The intrinsic pathway is activated by the DENV membrane protein ectodomain export from golgi to plasma membrane^59^. The NS2b-NS3 protease precursor and NS3 protease induce apoptosis^60^ most likely through the caspase-8 pathway^61^ or NF-κB^62^.

### Innate immunity associated with severe disease

Some immune responses are implicated with disease severity. TLR4 recognition of NS1 leads to pro-inflammatory cytokine production that contribute to vascular damage^63^ (Fig. 3). NS1 will also exacerbate disease by binding to uninfected cells to initiate vascular leakage^64,65^. Activation of the alternative complement pathway is associated with disease severity^66^. Apoptosis may contribute to disease severity; apoptotic cells were found in liver, cerebral, and endothelial cells from autopsies of patients with dengue hemorrhagic fever (DHF)/dengue shock syndrome^67^.

DCs are one of the first immune cells that encounter DENV following infection and send signals to recruit NK cells via type I IFN and TNF-α, resulting in perforin/granzyme activity, Fas/Fas ligand-dependent virus killing, and IFNγ production^68^. It is unclear if increased number of NK cells contribute to increase in disease severity. KIR3DLI, an inhibitory receptor of the killer immunoglobulin-like receptor (KIR) family that is expressed on NK cells, binds to DENV NS1^69^ and is associated with development of DHF. However, acutely infected patients in Thailand showed no correlation between NK cell subsets and level of severity^70^.

## Innate immune evasion

### Inhibition and evasion of type I IFN response

**Fig. 4:**
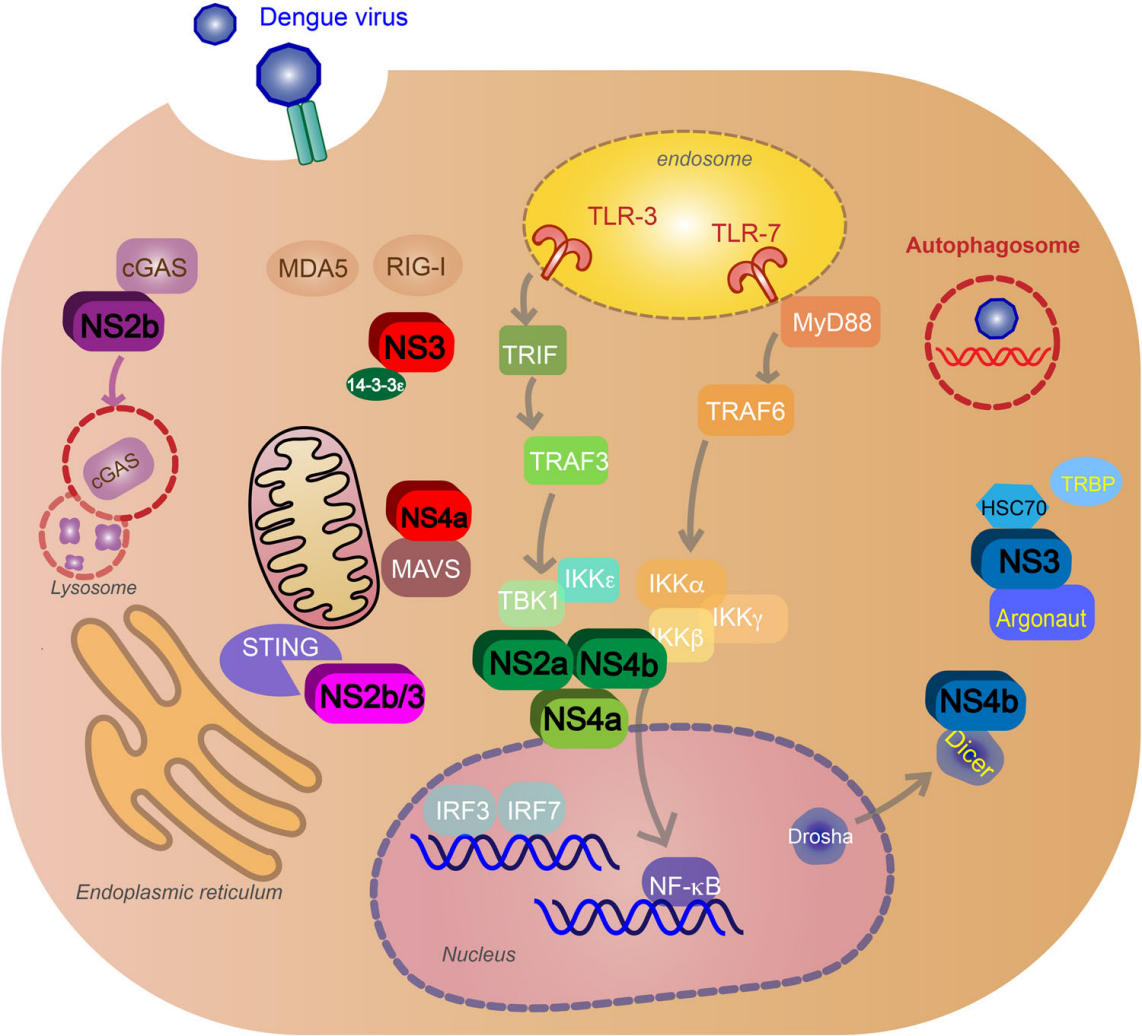
DENV evasion of innate immune response. Nonstructural proteins block signaling pathways after virus recognition and inhibit type I IFN production. NS3 and NS4a block retinoic acid-inducible gene I (RIG-I) translocation to mitochondria (red). NS2a and NS4b from DENV1, -2, -4 (green) and NS4a from DENV1 (light green) inhibit the activation of TANK-binding kinase 1 (TBK1), blocking the RIG-I/mitochondrial antiviral signaling (MAVS) signaling pathway and IFNβ induction. NS2b targets cyclic GMP-AMP synthase (cGAS) for autophagy–lysosome-dependent degradation and prevents mitochondrial DNA sensing (purple). NS2b/3 protease inhibits IFN production by cleaving stimulator of interferon gene (STING) (pink). NS4b inhibits RNAi by binding to Dicer, preventing the biogenesis of miRNA. NS3 will also block RNAi by binding to heat-shock cognate 70, an essential protein for creating RISC, and inhibit TRBP–Argonaute interaction and RISC formation (blue). DENV will exploit the autophagy pathway and use autophagosomes for replication, assembly and maturation, and evasion of neutralizing antibodies during transmission (red)

DENV uses NS proteins to block or inhibit signaling pathways in the infected cells. It also can block pathways that alert nearby cells of infection. DENV can block PRR signaling and production of type I IFN response by targeting the RLR and TLR pathways mentioned above. NS5 2′-O-methylation of 5′ prevents the virus from being sensed by RIG-I^71^. NS3 blocks RIG-I translocation to mitochondria by binding with the mitochondrial-targeting chaperone protein 14-3-3ε^72^. NS4a will also inhibit RIG-I interaction with MAVS by binding to the MAVS CARD-like domain and transmembrane domain^73^. NS2a and NS4b from DENV1, 2, 4, and NS4a from DENV1 block the RIG-I/MAVS signaling pathway by preventing phosphorylation of TBK1/IRF, inhibiting IFNβ induction^74^. NS2b targets cGAS for autophagy–lysosome-dependent degradation and prevents mitochondrial DNA sensing^38^. NS2b/3 protease inhibits IFN production by cleaving STING^75^ (Fig. 4). NS4b triggers elongation of mitochondria by inactivating mitochondrial fission factor dynamin-related protein 1, resulting in altered mitochondria-associated membranes (MAMs), increased DENV replication^76^, and decreased IFN production—possibly by blocking activated RIG-I recruitment to MAMs^77^.

**Fig. 5:**
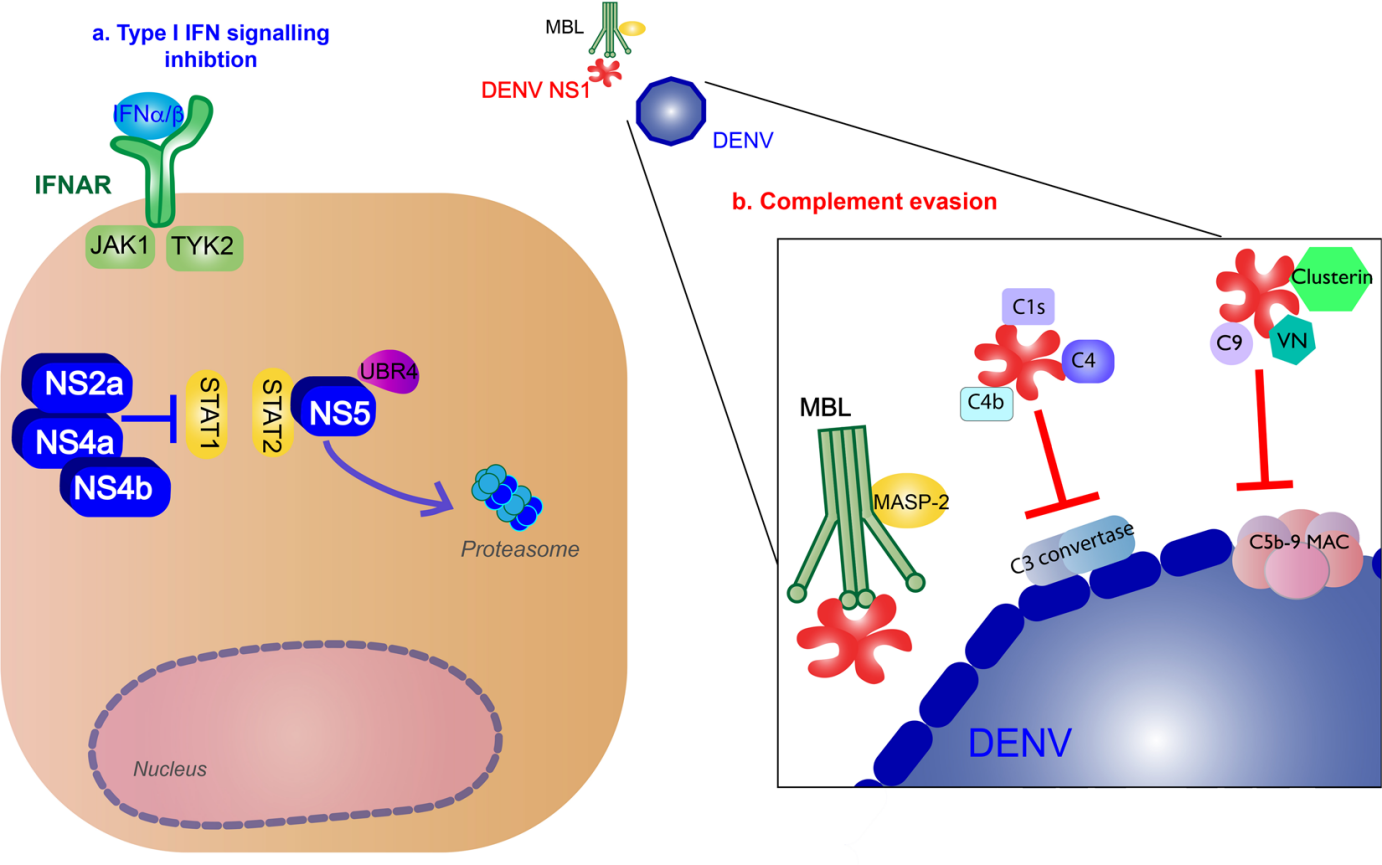
DENV inhibition of type I IFN response and complement pathways. **a** Type I IFN signaling inhibition (blue): NS2a, NS4a, and NS4b complex inhibit signal transducer and activator of transcription 1 (STAT1) signaling after IFNα/β receptor activation. NS5 will cause proteasomal degradation of STAT2 by binding with the host ubiquitin protein ligase E3 component N-recognin 4 (UBR4). **b** Complement evasion (red): NS1 hexamer inhibits the formation of the classical pathway C3 convertase by binding to C4, C1s, and C4b. NS1 inhibits neutralization by the mannose-binding lectin (MBL) pathway by binding to MBL. NS1 blocks membrane attack complex (MAC) formation by binding to complement regulators vitronectin (VN), C9, or clusterin (Clu)

Furthermore, along with inhibiting IFNα/β production, NS proteins block the IFNAR pathways. NS2a, NS4a, and NS4b complex is responsible for subverting the IFNα/β response by inhibiting STAT1 signaling after IFNAR activation in vitro^78^. NS5 inhibits IFN-mediated response via STAT2 by binding with the host ubiquitin protein ligase E3 component N-recognin 4 for proteasomal degradation of STAT2^79^ (Fig. 5).

### Other immune evasion mechanisms

DENV will subvert apoptosis early in the life cycle to ensure viral replication. The phosphatidylinositol 3 kinase/Akt (PI3K/Akt) pathway plays an important role in cell survival, regulating proliferation, and inhibiting apoptosis; many viruses modulate this pathway for survival during early, chronic, and latent infections^80^. DENV2 blocks caspase-dependent apoptosis in cells during early infection by activating the PI3K/Akt pathway^81^.

The virus evades the complement response using NS1. NS1 inhibits the formation of the classical pathway C3 convertase by binding to C4, C1s, and C4b^82^. NS1 also protects DENV from neutralization by binding to MBL^83^. To block the formation of MAC, NS1 will bind to complement regulators vitronectin or inhibit C9 polymerization^84^. NS1 binding to complement inhibitory factor clusterin will also inhibit MAC formation^85^ (Fig. 5).

Other DENV proteins have been shown to have immune evasion activity. NS4b suppresses the RNAi pathway in mammalian cells by blocking Dicer processing via transmembrane domain 3 (TMD3) and TMD5, preventing the biogenesis of mRNA; this is independent of the N-terminal domain that is associated with IFN suppression^48^. NS3 will also block RNAi by binding to heat-shock cognate 70, an essential protein for creating RISC, and inhibit TRBP–Argo interaction and RISC formation^86^ (Fig. 4). DENV NS2b/3 protease inhibits reticulophagy (selective autophagy of the ER) by cleaving *FAM134B*, an essential receptor for reticulophagy^53^. DENV C protein aids in inhibition of apoptosis in Huh7 cells by interacting with calcium-modulating cyclophilin-binding ligand, an ER protein associated with cell survival by regulating Bim-dependent death^87^. DENV C also interacts with cellular protein DEAD (Asp-Glu-Ala-Asp) Box Helicase 3, X-linked, resulting in higher viral titers and down-regulation of innate immune responses against DENV independent of the type I IFN pathway^88^.

## Future directions

There is currently one licensed vaccine, Dengvaxia by Sanofi-Pasteur, which uses the prM-E dengue sequence in a yellow fever virus backbone. However, the administration of the vaccine has been widely criticized, since it has only about 60% efficacy^89^. There are no approved antivirals for DENV. It is necessary to understand the role of the innate immune response to DENV in order to produce an effective vaccine or antiviral against DENV. Treatment for clinical manifestations of the acute febrile are paracetamol for high fever and oral or intravenous fluid intake^90^.

Recent DENV antiviral research has been focusing on identifying novel compounds that targeting the DENV proteins responsible for replication and innate immune evasion. A compound targeting NS4b, NITD-618, inhibits RNA synthesis in vitro in all four serotypes of DENV^91^. The novel small-molecule compound, ST-147, inhibits DENV replication by targeting the capsid protein and reduced viremia and viral load in mice^92^. Two compounds targeting protease and RdRp activity show protection from DENV2 in mice, with the combination of the compounds having synergistic inhibition in vitro against DENV2^93^. There are many potential NS2B/NS3 protease targets that reduce DENV infectivity in vitro^94^. Additionally, a therapeutic monoclonal antibody raised against NS1 induced complement-mediated lysis in vitro and had protective effects in vivo^95^.

Drug repurposing have been investigated for DENV inhibition. Minocycline, typically used as an antibiotic and anti-inflammatory, inhibits DENV replication in vitro by suppressing migration inhibitory facto, a catalyst for autophagy, and this drug also decreased viremia and autophagy formation in vivo^96^. AR-12, a derivative of celecoxib (a non-steroidal anti-inflammatory drug) that inhibits PI3K/Akt signaling (the pathway that has shown to be activated by DENV2 to block apoptosis^97^), reduces viral replication in all four serotypes in vitro and protects against DEV2 in vivo^81^. Schisandrin A, a derivative of the herbal medicinal plant *Schisandra chinesis*, inhibited DENV replication and increased type I IFN response in vitro and in vivo^98^. Secreted phospholipase A_2_, obtained from snake venom showed viricidal, has neutralizing activity against DENV, targeting the virus envelope lipid bilayer^99^.

## Conclusion

DENV is a mosquito-borne *Flavivirus* that is endemic in many tropical and sub-tropical countries. The NS proteins are responsible for viral replication and host innate immune evasion. The innate immune response to DENV is not well characterized nor are the exact roles of the NS proteins in evading the immune response. The main innate immune response is type I IFN and the main evasion mechanism of the virus is to target against the type I IFN response. Other innate immune responses include complement activation, apoptosis, autophagy, and RNAi. It is important to understand the host innate immune response to infection and how the virus evades or exploits this in order to develop effective antivirals and vaccines.

## References

[1] Brady OJ (2012). Refining the global spatial limits of dengue virus transmission by evidence-based consensus. PLoS Negl. Trop. Dis..

[2] Messina JP (2014). Global spread of dengue virus types: mapping the 70 year history. Trends Microbiol..

[3] Thomas Stephen J., Endy Timothy P., Rothman Alan L. (2014). Flaviviruses: Dengue. Viral Infections of Humans.

[4] Screaton G, Mongkolsapaya J, Yacoub S, Roberts C (2015). New insights into the immunopathology and control of dengue virus infection. Nat. Rev. Immunol..

[5] Halstead Saacute, Nimmannitya S, Cohen S (1970). Observations related to pathogenesis of dengue hemorrhagic fever. IV. Relation of disease severity to antibody response and virus recovered. Yale J. Biol. Med..

[6] Littaua R, Kurane I, Ennis F (1990). Human IgG Fc receptor II mediates antibody-dependent enhancement of dengue virus infection. J. Immunol..

[7] Chambers TJ, Hahn CS, Galler R, Rice CM (1990). Flavivirus genome organization, expression, and replication. Annu. Rev. Microbiol..

[8] Kuhn RJ (2002). Structure of dengue virus: implications for flavivirus organization, maturation, and fusion. Cell.

[9] Zhang Y (2003). Structures of immature flavivirus particles. EMBO J..

[10] Zhang W (2003). Visualization of membrane protein domains by cryo-electron microscopy of dengue virus. Nat. Struct. Mol. Biol..

[11] Winkler G, Randolph VB, Cleaves GR, Ryan TE, Stollar V (1988). Evidence that the mature form of the flavivirus nonstructural protein NS1 is a dimer. Virology.

[12] Flamand M (1999). Dengue virus type 1 nonstructural glycoprotein NS1 is secreted from mammalian cells as a soluble hexamer in a glycosylation-dependent fashion. J. Virol..

[13] Lindenbach BD, Rice CM (1999). Genetic interaction of flavivirus nonstructural proteins NS1 and NS4A as a determinant of replicase function. J. Virol..

[14] Watterson D, Modhiran N, Young PR (2016). The many faces of the flavivirus NS1 protein offer a multitude of options for inhibitor design. Antivir. Res..

[15] Scaturro P, Cortese M, Chatel-Chaix L, Fischl W, Bartenschlager R (2015). Dengue virus non-structural protein 1 modulates infectious particle production via interaction with the structural proteins. PLoS Pathog..

[16] Miller S, Kastner S, Krijnse-Locker J, Bühler S, Bartenschlager R (2007). The non-structural protein 4A of dengue virus is an integral membrane protein inducing membrane alterations in a 2K-regulated manner. J. Biol. Chem..

[17] Xie X, Zou J, Puttikhunt C, Yuan Z, Shi PY (2015). Two distinct sets of NS2A molecules are responsible for dengue virus RNA synthesis and virion assembly. J. Virol..

[18] Falgout B, Pethel M, Zhang Y, Lai C (1991). Both nonstructural proteins NS2B and NS3 are required for the proteolytic processing of dengue virus nonstructural proteins. J. Virol..

[19] Umareddy I, Chao A, Sampath A, Gu F, Vasudevan SG (2006). Dengue virus NS4B interacts with NS3 and dissociates it from single-stranded RNA. J. Gen. Virol..

[20] Issur M (2009). The flavivirus NS5 protein is a true RNA guanylyltransferase that catalyzes a two-step reaction to form the RNA cap structure. RNA.

[21] Cruz-Oliveira C (2015). Receptors and routes of dengue virus entry into the host cells. FEMS Microbiol. Rev..

[22] Garcia M, Wehbe M, Lévêque N, Bodet C (2017). Skin innate immune response to flaviviral infection. Eur. Cytokine Netw..

[23] Heinz FX, Allison SL (2000). Structures and mechanisms in flavivirus fusion. Adv. Virus Res..

[24] Mackenzie J (2005). Wrapping things up about virus RNA replication. Traffic.

[25] Polacek C, Friebe P, Harris E (2009). Poly (A)-binding protein binds to the non-polyadenylated 3′ untranslated region of dengue virus and modulates translation efficiency. J. Gen. Virol..

[26] Welsch S (2009). Composition and three-dimensional architecture of the dengue virus replication and assembly sites. Cell Host Microbe.

[27] Mukhopadhyay S, Kuhn RJ, Rossmann MG (2005). A structural perspective of the flavivirus life cycle. Nat. Rev. Microbiol..

[28] Junjhon J (2010). Influence of pr-M cleavage on the heterogeneity of extracellular dengue virus particles. J. Virol..

[29] Dejnirattisai W (2010). Cross-reacting antibodies enhance dengue virus infection in humans. Science.

[30] Loo YM, Gale M (2011). Immune signaling by RIG-I-like receptors. Immunity.

[31] Akira S, Takeda K (2004). Toll-like receptor signalling. Nat. Rev. Immunol..

[32] Nasirudeen A (2011). RIG-I, MDA5 and TLR3 synergistically play an important role in restriction of dengue virus infection. PLoS Negl. Trop. Dis..

[33] Wang JP (2006). Flavivirus activation of plasmacytoid dendritic cells delineates key elements of TLR7 signaling beyond endosomal recognition. J. Immunol..

[34] Seth RB, Sun L, Ea CK, Chen ZJ (2005). Identification and characterization of MAVS, a mitochondrial antiviral signaling protein that activates NF-κB and IRF3. Cell.

[35] Baum A, García-Sastre A (2010). Induction of type I interferon by RNA viruses: cellular receptors and their substrates. Amino Acids.

[36] Sun, B. et al. Dengue virus activates cGAS through the release of mitochondrial DNA. *Sci. Rep*. **7**, 3594 (2017). 10.1038/s41598-017-03932-1 PMC5472572 28620207

[37] El-Bacha T (2007). Mitochondrial and bioenergetic dysfunction in human hepatic cells infected with dengue 2 virus. Biochim. Biophys. Acta.

[38] Aguirre S (2017). Dengue virus NS2B protein targets cGAS for degradation and prevents mitochondrial DNA sensing during infection. Nat. Microbiol..

[39] Wu J (2013). Cyclic GMP-AMP is an endogenous second messenger in innate immune signaling by cytosolic DNA. Science.

[40] Lai Jenn‐Haung, Wang Mei‐Yi, Huang Chuan‐Yueh, Wu Chien‐Hsiang, Hung Li‐Feng, Yang Chia‐Ying, Ke Po‐Yuan, Luo Shue‐Fen, Liu Shih‐Jen, Ho Ling‐Jun (2018). Infection with the dengue RNA virus activates TLR9 signaling in human dendritic cells. EMBO reports.

[41] Diamond MS (2000). Modulation of dengue virus infection in human cells by alpha, beta, and gamma interferons. J. Virol..

[42] Morrison J, García-Sastre A (2014). STAT2 signaling and dengue virus infection. JAK-STAT.

[43] Avirutnan P (2011). Complement-mediated neutralization of dengue virus requires mannose-binding lectin. MBio.

[44] Thiel S (1997). A second serine protease associated with mannan-binding lectin that activates complement. Nature.

[45] Fujita T, Matsushita M, Endo Y (2004). The lectin‐complement pathway—its role in innate immunity and evolution. Immunol. Rev..

[46] Sánchez-Vargas I (2009). Dengue virus type 2 infections of Aedes aegypti are modulated by the mosquito’s RNA interference pathway. PLoS Pathog..

[47] Jeang KT (2012). RNAi in the regulation of mammalian viral infections. BMC Biol..

[48] Kakumani PK (2013). Role of RNA interference (RNAi) in dengue virus replication and identification of NS4B as an RNAi suppressor. J. Virol..

[49] Urcuqui-Inchima Silvio, Cabrera Jesús, Haenni Anne-Lise (2017). Interplay between dengue virus and Toll-like receptors, RIG-I/MDA5 and microRNAs: Implications for pathogenesis. Antiviral Research.

[50] Kuma A (2004). The role of autophagy during the early neonatal starvation period. Nature.

[51] Mizushima N, Levine B, Cuervo AM, Klionsky DJ (2008). Autophagy fights disease through cellular self-digestion. Nature.

[52] Panyasrivanit M (2011). Induced autophagy reduces virus output in dengue infected monocytic cells. Virology.

[53] Lennemann NJ, Coyne CB (2017). Dengue and Zika viruses subvert reticulophagy by NS2B3-mediated cleavage of FAM134B. Autophagy.

[54] Lee YR (2008). Autophagic machinery activated by dengue virus enhances virus replication. Virology.

[55] Mateo R (2013). Inhibition of cellular autophagy deranges dengue virion maturation. J. Virol..

[56] Wu YW (2016). Autophagy-associated dengue vesicles promote viral transmission avoiding antibody neutralization. Sci. Rep..

[57] Elmore S (2007). Apoptosis: a review of programmed cell death. Toxicol. Pathol..

[58] Netsawang J (2010). Nuclear localization of dengue virus capsid protein is required for DAXX interaction and apoptosis. Virus Res..

[59] Catteau A (2003). Dengue virus M protein contains a proapoptotic sequence referred to as ApoptoM. J. Gen. Virol..

[60] Shafee N, AbuBakar S (2003). Dengue virus type 2 NS3 protease and NS2B-NS3 protease precursor induce apoptosis. J. Gen. Virol..

[61] Ramanathan MP (2006). Host cell killing by the West Nile Virus NS2B–NS3 proteolytic complex: NS3 alone is sufficient to recruit caspase-8-based apoptotic pathway. Virology.

[62] Lin, J.-C. et al. Dengue viral protease interaction with NF-kB inhibitor a/b results in endothelial cell apoptosis and hemorrhage development. **193**, 1258–1267 (2014). 10.4049/jimmunol.1302675 24973451

[63] Modhiran N (2015). Dengue virus NS1 protein activates cells via Toll-like receptor 4 and disrupts endothelial cell monolayer integrity. Sci. Transl. Med..

[64] Avirutnan P (2006). Vascular leakage in severe dengue virus infections: a potential role for the nonstructural viral protein NS1 and complement. J. Infect. Dis..

[65] Avirutnan P (2007). Secreted NS1 of dengue virus attaches to the surface of cells via interactions with heparan sulfate and chondroitin sulfate E. PLoS Pathog..

[66] Nascimento EJ (2009). Alternative complement pathway deregulation is correlated with dengue severity. PLoS ONE.

[67] Limonta D, Capó V, Torres G, Pérez AB, Guzmán MG (2007). Apoptosis in tissues from fatal dengue shock syndrome. J. Clin. Virol..

[68] Costa VV (2017). Dengue virus-infected dendritic cells, but not monocytes, activate natural killer cells through a contact-dependent mechanism involving adhesion molecules. mBio.

[69] Townsley E., O'Connor G., Cosgrove C., Woda M., Co M., Thomas S. J., Kalayanarooj S., Yoon I.-K., Nisalak A., Srikiatkhachorn A., Green S., Stephens H. A. F., Gostick E., Price D. A., Carrington M., Alter G., McVicar D. W., Rothman A. L., Mathew A. (2015). Interaction of a dengue virus NS1-derived peptide with the inhibitory receptor KIR3DL1 on natural killer cells. Clinical & Experimental Immunology.

[70] Keawvichit, R. et al. Differences in activation and tissue homing markers of natural killer cell subsets during acute dengue infection. *Immunology* **153**, 455–465 (2017). 10.1111/imm.12858 PMC5838507 29105052

[71] Chang DC (2016). Evasion of early innate immune response by 2′-O-methylation of dengue genomic RNA. Virology.

[72] Chan YK, Gack MU (2016). A phosphomimetic-based mechanism of dengue virus to antagonize innate immunity. Nat. Immunol..

[73] He Z (2016). Dengue virus subverts host innate immunity by targeting adaptor protein MAVS. J. Virol..

[74] Dalrymple NA, Cimica V, Mackow ER (2015). Dengue virus NS proteins inhibit RIG-I/MAVS signaling by blocking TBK1/IRF3 phosphorylation: dengue virus serotype 1 NS4A is a unique interferon-regulating virulence determinant. MBio.

[75] Yu CY (2012). Dengue virus targets the adaptor protein MITA to subvert host innate immunity. PLoS Pathog..

[76] Barbier V, Lang D, Valois S, Rothman AL, Medin CL (2017). Dengue virus induces mitochondrial elongation through impairment of Drp1-triggered mitochondrial fission. Virology.

[77] Chatel-Chaix L (2016). Dengue virus perturbs mitochondrial morphodynamics to dampen innate immune responses. Cell Host Microbe.

[78] Muñoz-Jordán JL, Sánchez-Burgos GG, Laurent-Rolle M, García-Sastre A (2003). Inhibition of interferon signaling by dengue virus. Proc. Natl. Acad. Sci. USA.

[79] Morrison J (2013). Dengue virus co-opts UBR4 to degrade STAT2 and antagonize type I interferon signaling. PLoS Pathog..

[80] Cooray S (2004). The pivotal role of phosphatidylinositol 3-kinase–Akt signal transduction in virus survival. J. Gen. Virol..

[81] Chen HH (2017). AR-12 suppresses dengue virus replication by down-regulation of PI3K/AKT and GRP78. Antivir. Res..

[82] Avirutnan, P. et al. Antagonism of the complement component C4 by flavivirus nonstructural protein NS1. *J. Exp. Med.* 20092545 (2010). 10.1084/jem.20092545 PMC2856034 20308361

[83] Thiemmeca S (2016). Secreted NS1 protects dengue virus from mannose-binding lectin-mediated neutralization. J. Immunol..

[84] Conde JN (2016). Inhibition of the membrane attack complex by dengue virus NS1 through interaction with vitronectin and terminal complement proteins. J. Virol..

[85] Kurosu T, Chaichana P, Yamate M, Anantapreecha S, Ikuta K (2007). Secreted complement regulatory protein clusterin interacts with dengue virus nonstructural protein 1. Biochem. Biophys. Res. Commun..

[86] Kakumani PK (2015). Dengue NS3, an RNAi suppressor, modulates the human miRNA pathways through its interacting partner. Biochem. J..

[87] Li J (2012). Dengue virus utilizes calcium modulating cyclophilin-binding ligand to subvert apoptosis. Biochem. Biophys. Res. Commun..

[88] Kumar R, Singh N, Abdin MZ, Patel AH, Medigeshi GR (2017). Dengue virus capsid interacts with DDX3X–a potential mechanism for suppression of antiviral functions in dengue infection. Front. Cell. Infect. Microbiol..

[89] Aguiar M, Stollenwerk N, Halstead SB (2016). The risks behind Dengvaxia recommendation. Lancet Infect. Dis..

[90] Organization, W. H. et al. *Dengue: Guidelines for Diagnosis, Treatment, Prevention and Control* (World Health Organization, Geneva, 2009). 23762963

[91] Xie X (2011). Inhibition of dengue virus by targeting viral NS4B protein. J. Virol..

[92] Byrd CM (2013). A novel inhibitor of dengue virus replication that targets the capsid protein. Antimicrob. Agents Chemother..

[93] Pelliccia S (2017). Inhibition of dengue virus replication by novel inhibitors of RNA-dependent RNA polymerase and protease activities. J. Enzym. Inhib. Med. Chem..

[94] Wu H (2015). Novel dengue virus NS2B/NS3 protease inhibitors. Antimicrob. Agents Chemother..

[95] Wan SW (2017). Therapeutic effects of monoclonal antibody against dengue virus NS1 in a STAT1 knockout mouse model of dengue infection. J. Immunol..

[96] Lai Yen-Chung, Chuang Yung-Chun, Chang Chih-Peng, Lin Yee-Shin, Perng Guey-Chuen, Wu Han-Chung, Hsieh Shie-Liang, Yeh Trai-Ming (2018). Minocycline suppresses dengue virus replication by down-regulation of macrophage migration inhibitory factor-induced autophagy. Antiviral Research.

[97] Lee CJ, Liao CL, Lin YL (2005). Flavivirus activates phosphatidylinositol 3-kinase signaling to block caspase-dependent apoptotic cell death at the early stage of virus infection. J. Virol..

[98] Yu, J.-S. et al. Schisandrin A inhibits dengue viral replication via upregulating antiviral interferon responses through STAT signaling pathway. *Sci. Rep.* **7** 45171 (2017). 10.1038/srep45171 PMC5364541 28338050

[99] Aoki-Utsubo C (2017). Broad-spectrum antiviral agents: secreted phospholipase A 2 targets viral envelope lipid bilayers derived from the endoplasmic reticulum membrane. Sci. Rep..

[100] He L, Hannon GJ (2004). MicroRNAs: small RNAs with a big role in gene regulation. Nat. Rev. Genet..

